# Fabrication of Human Milk Fat Substitute: Based on the Similarity Evaluation Model and Computer Software

**DOI:** 10.3390/molecules29092096

**Published:** 2024-05-01

**Authors:** Huiquan Zhu, Pu Zhao, Xiaodan Wang, Yunna Wang, Shuwen Zhang, Xiaoyang Pang, Jiaping Lv

**Affiliations:** 1 Institute of Food Science and Technology, Chinese Academy of Agricultural Sciences, Beijing 100193, China; 13834083105@163.com (H.Z.); hscaas@163.com (P.Z.); prettyshoot@126.com (X.W.); wang_yn92@163.com (Y.W.); zswcaas@hotmail.com (S.Z.); lvjiapingcaas@126.com (J.L.); 2 Laboratory of Chemistry of Natural Molecules, Gembloux Agro-Bio Tech, University of Liege, 5030 Gembloux, Belgium; 3 National Center of Technology Innovation for Dairy, Hohhot 010100, China

**Keywords:** vegetable oils, evaluation model, software, in vivo experiment, intestinal flora

## Abstract

We aimed to obtain the optimal formula for human milk fat substitute (HMFS) through a combination of software and an evaluation model and further verify its practicability through an animal experiment. The results showed that a total of 33 fatty acid (FA) and 63 triglyceride (TAG) molecular species were detected in vegetable oils. Palmitic acid, oleic acid, linoleic acid, 18:1/16:0/18:1, 18:2/16:0/18:2, 18:1/18:1/18:1 and 18:1/18:2/18:1, were the main molecular species among the FAs and TAGs in the vegetable oils. Based on the HMFS evaluation model, the optimal mixed vegetable oil formula was blended with 21.3% palm oil, 2.8% linseed oil, 2.6% soybean oil, 29.9% rapeseed oil and 43.4% maize oil, with the highest score of 83.146. Moreover, there was no difference in the weight, blood routine indices or calcium and magnesium concentrations in the feces of the mice between the homemade mixed vegetable oil (HMVO) group and the commercial mixed vegetable oil (CMVO) group, while nervonic acid (C24:1) and octanoic acid (C8:0) were absorbed easily in the HMVO group. Therefore, these results demonstrate that the mixing of the different vegetable oils was feasible via a combination of computer software and an evaluation model and provided a new way to produce HMFS.

## 1. Introduction

Human milk fat (HMF) is constructed with a core composed of triacylglycerol (TAG) and a membrane (mainly consisting of phospholipid and protein). The TAG content accounts for about 98% of the total HMF, as it is important for the growth of newborns [1]. More than 400 TAG molecular species are detected in HMF [2], in which oleic acid–palmitic acid–oleic acid (OPO) and oleic acid–palmitic acid–linoleic acid (OPL) are the main molecular species, accounting for 11.13–28.08% and 8.86–19.50% of the total TAGs, respectively [3,4,5,6]. TAGs are impacted by some factors, such as the lactation period, the region sampled, the physical condition of the voluntary mothers dietary patterns, etc. Most studies have found that the content of some TAG molecular species, such as OPO and OPL, decreases with the prolongation of lactation time [3,6]. Chen et al. (2020) reported that the content of OPL was the highest (16.55%, 19.20% and 18.67%, respectively) in breast milk samples from Zhengzhou, Wuhan and Harbin, followed by OPO (10.08, 10.22 and 12.03%, respectively) [7].

Infant formula (IF) manufacturers have been committed to meeting the nutrition needs of infants during their growth stage, so HMF substitute (HMFS) is used in IF production, which is based on the composition of HMF and selected from many plant, animal and microbial oils or fats mixed scientifically [8]. There are two main kinds of HMFS; the first arises from a variety of oil-compounding methods to adjust the fatty acid (FA) composition of baby powder, among which vegetable oil and cow milk fat are the most common sources [9]. Vegetable oils are economical and contain different FA and TAG molecular species, so they can safely mimic HMF and meet the lipid needs of infants through physical mixing. Wei et al. (2019) reported that there are 48 TAG molecular species detected in all vegetable oils, and they showed the different characteristics of TAGs among vegetable oils; for example, OPO and OOO accumulated in olive oil and linseed oil had more linolenic acid–linolenic acid–linolenic acid or linolenic acid–linoleic acid–linolenic acid [10]. The second comprises structural lipids, mainly containing OPO-structured TAGs and other structured lipids enriched with palmitic acid (C16:0) in the Sn-2 position, and they are obtained by transesterification [11]. Ghide et al. (2022) used palmitic acid–palmitic acid–palmitic acid as a substrate to synthesize OPO through rhizomucor miehei lipase, in which the percentage of Sn-2 C16:0 and Sn-1,3 oleic acid (Sn-1,3 C18:1) reached 92.93% and 57.82%, respectively [12]. 

Previous studies have shown that the intestinal microbial composition is affected by the FA types and TAG structure of HMFS [13]. Chen et al. (2022) carried out a 16S rRNA gene sequence analysis and metabolomics analysis, and the results showed that the abundance of bifidobacterium increased after the feeding of OPO-containing formula, which was likely due to the adhesion and proliferation of lactobacillus, and Bifidobacterium was directly affected by mono-triglyceride palmitate at the Sn-2 position [14,15]. For the influence on the intestinal flora caused by the mixed vegetable oils, Qiao et al. (2022) found that the abundances of Escherichia coli, Lactobacilli and bifidobacteria decreased after adding mixed vegetable oils to the daily diet of mice; meanwhile, the activity of protease, cellulase and amylase also decreased [16]. 

The model used to evaluate the similarity between HMF and HMFS was first established by Wang et al. (2010) to analyze the similarity between FAs and Sn-2 FAs in HMF and HMFS based on the reduction principle, and Hokkanen et al. (2022) reported another evaluation model based on the Bray–Curtis index [17,18]. In this study, according to the human milk data reported in our previous study [3], software based on C++ was set up, and it was used to calculate the different formulas of mixed vegetable oils according to the evaluation model. After obtaining the optimal formula, it was used for an in vivo experiment with commercial mixed vegetable oil to evaluate the intestinal flora of mice and further verify the practicability of the software. These results will provide a new approach to obtaining different formulas for HMFS and further promote the development of HMFS.

## 2. Results and Discussion

### 2.1. Fatty Acid Profile of Vegetable Oils

A total of 33 FA species were detected in seven different vegetable oils (Table 1), including 16 saturated FAs (SFAs) and 17 unsaturated FAs (UFAs). The highest number of FA species was observed in rapeseed oil and walnut oil, in both of which 28 FA species were found, followed by soybean oil (27 FA species), palm oil (26 FA species), maize oil (25 FA species), linseed oil (24 FA species) and sunflower seed oil (22 FA species). Among these FA species, C16:0 (4.670–37.093% of the total FAs), C18:1n9c (18.917–53.225% of the total FAs), linoleic acid (C18:2n6c, 10.704–61.268% of the total FAs) and linolenic acid (C18:3n6, 0.142–44.235% of the total FAs) were the main species. It was evident that C16:0, C18:1n9c, C18:2n6c and C18:3n6 showed the highest percentages in palm oil, rapeseed oil, sunflower oil and linseed oil, respectively, accounting for 37.093% of the total FAs, 53.225% of the total FAs, 61.268% of the total FAs and 44.235% of the total FAs, respectively, which are in accordance with previous results [19,20,21,22,23]. Moreover, some FA species showed specificity in vegetable oils; for example mecilenic acid (C14:1) was only found in palm oil, and docosanoic acid (C22:0) was only found in rapeseed oil. For polyunsaturated fatty acids (PUFAs), such as docosahexaenoic acid (DHA, C22:6n3) and nervonic acid (C24:1n9), which are important for the brain development of infants [24], small concentrations of these FAs were found in the vegetable oils. C24:1n9 was only detected in walnut oil (0.038% of the total FAs) and rapeseed oil (0.141% of the total FAs), and DHA was found in palm oil, walnut oil and maize oil, accounting for 0.078% of the total FAs, 0.113% of the total FAs and 0.026% of the total FAs, respectively.

### 2.2. Triacylglycerol Profile of Vegetable Oils

The stereochemical structure of TAGs is important for the properties of oil, including its absorbing and utilizable characteristics. Based on the ultraperformance liquid chromatogram–mass spectrum (UPLC-MS) method, 63 different TAG molecular species were detected in all the vegetable oils (Table 2), and 60 TAG molecular species were detected in palm oil, followed by walnut oil (56), rapeseed oil (55), linseed oil (47), sunflower seed oil (45), soybean oil (44) and maize oil (44). Among these TAG molecular species, 18:1/16:0/18:1 (OPO), 18:2/16:0/18:2 (LPL), 18:1/18:1/18:1 (OOO), 18:1/18:2/18:1 (OLO), 18:2/18:0/18:2 (LSL), 18:2/18:1/18:2 (LOL) and 18:2/18:2/18:2 (LLL) were the main TAG molecular species, constituting 2.841–21.965% of the total TAGs, 1.088–12.538% of the total TAGs, 2.690–26.833% of the total TAGs, 1.058–22.025% of the total TAGs, 0.672–11.598% of the total TAGs, 0.181–16.181% of the total TAGs and 0.070–20.694% of the total TAGs, respectively. Similar results were reported in a previous study [10]. Moreover, we could see that the OPO percentage in palm oil was the highest, which was 21.965% of the total TAGs. The most abundant contents of OOO and OLO were both observed in rapeseed oil, accounting for 26.833% and 22.025% of the total TAGs, respectively. LLL showed the highest content (20.694% of total TAGs) in the sunflower seed oil. These results have also been reported by other researchers [25,26]. The percentage of 18:1/16:0/18:2 (OPL), another important TAG molecular species in human milk, accounted for 2.709–7.778% of the total TAGs in the different vegetable oils [2,7]. Furthermore, just like FAs, the TAG molecular species also demonstrated specificity in the different vegetable oils; for example, 12:0/6:0/12:0, 12:0/8:0/12:0, 14:0/16:0/14:0, 16:0/12:0/16:0, 16:0/14:0/16:0 and 16:0/12:0/18:0 were only detected in palm oil. 

### 2.3. Formula of Mixed Vegetable Oils 

The software was input in Excel (Microsoft Office software 2016, Microsoft, USA) form, and the total results, including the top 1000 formulas, were also output in Excel form (Table S2). Table 3 showed the optimal mixing ratio result of the vegetable oils, which was simulated according to the evaluation model of HMF and scored with the reduction principle. The results showed that the highest score for the mixed vegetable oils was 83.146, which was mixed with 21.3% palm oil, 2.8% linseed oil, 2.6% soybean oil, 29.9% rapeseed oil and 43.4% maize oil (Table S2). There were some reasons why the highest score was not more than 90: first, some TAG molecular species, such as 18:0/16:0/18:1, 18:1/16:0/12:0, 18:1/16:0/14:0, 18:2/14:0/18:1, 18:1/16:0/16:1 and 18:1/12:0/18:2, only existed in HMF, leading to an inevitable score reduction for the mixed vegetable oils; furthermore, the deduction points for OPL and 18:0/14:0/18:1 were relatively higher. This finding might have been caused by the fact that the minimum concentrations of OPL and 18:0/14:0/18:1 in human milk were higher than those of all the vegetable oils.

### 2.4. Results of In Vivo Experiment

#### 2.4.1. Influence on the Growth, Blood Routine and Serum Biochemical Indices of Sprague-Dawley Mice

An in vivo experiment was used to verify the availability of the optimal formula made by the software and evaluation model. The experimental results showed that the body weight of the Sprague-Dawley (SD) mice increased by about 10 g/day in both the group administered homemade mixed vegetable oil obtained with the software mentioned above (HMVO) and the commercial mixed vegetable oil (CMVO) group, and their food utilization rate was 38–42% (Table S3), indicating that there was no significant difference in body weight and food intake between the SD mice in the HMVO and CMVO groups. A blood routine examination, a basic examination for disease, can reflect the pathological condition of various tissues and organs in the body [16]. It was evident that the contents of red blood cells (RBCs), hemoglobin (HGB), hematocrit (HCT), mean red blood cell hemoglobin (MCH), platelets (PLTs), white blood cells (WBCs), lymphocytes (LYMs), neutrophils (NEUTs), monocytes (MONOs), eosinophils (EOs), basophils (BAEOs) and reticulocytes (RETs), the mean red blood cell volume (MCV), the mean red blood cell hemoglobin concentration (MCHC), the serum total cholesterol (CHO), the total triglyceride (TG), the low-density lipoprotein cholesterol level (LDL-c) and the high-density lipoprotein cholesterol level (HDL-c) were not impacted in the HMVO and CMVO groups, indicating that the influence caused by the HMVO and the CMVO was similar (Table S4). 

#### 2.4.2. Fatty Acid Profile of Intestinal Contents and Feces

A total of 16 FA species were detected in the small intestine contents (Table S5). Among these FAs, the percentage of C16:0 was the highest, accounting for 30.86–32.68% of the total FAs, followed by stearic acid (C18:0, 24.46–25.70% of the total FAs), C18:3n6 (11.24–14.00% of the total FAs), C20:3 (9.56–13.61% of the total FAs) and C18:1n9c (9.03–12.30% of the total FAs). Furthermore, the contents of caprylic acid (C8:0), tetracosanoic acid (C24:0) and C24:1 in the SD mice in the HMVO group were all lower than those in the CMVO group (*p* < 0.05), as a consequence of the fact that C8:0 and C24:1 in the HMVO group might be absorbed more quickly by the SD mice. C24:1 has been verified to be related to the early development of premature infants, whose brain maturation is associated with the C24:1 concentration in sphingomyelin [27]. However, for the FA profile in the SD mice’s feces, we only found 11 FA species, among which butyric acid (C4:0), caproic acid (C6:0), C8:0, C18:1n9c and eicosatetraenoic acid (C20:4n6) were significantly influenced (*p* < 0.05). The percentages of C4:0, C6:0, C8:0 and C20:4n6 in the SD mice in the HMVO group were significantly lower than those in the CMVO group, while the C18:1n9c concentration in the CMVO group was higher, which indicates that the HMVO could promote the absorption of these FAs mentioned above.

#### 2.4.3. Concentration of Calcium and Magnesium in Feces

Because calcium and magnesium ions were combined with free FAs to form calcium soap in the intestine, the concentrations of calcium and magnesium were detected in this research (Table S6). The results showed that the concentrations of calcium and magnesium were 9.72 mg/g and 0.54 mg/g in the HMVO group, respectively, and both were lower than those in the CMVO group (calcium: 10.05 mg/g; magnesium: 0.60 mg/g).

### 2.5. The Influence on Intestinal Flora

#### 2.5.1. Alpha (α) and Beta Diversity Analysis

**Figure 1:**
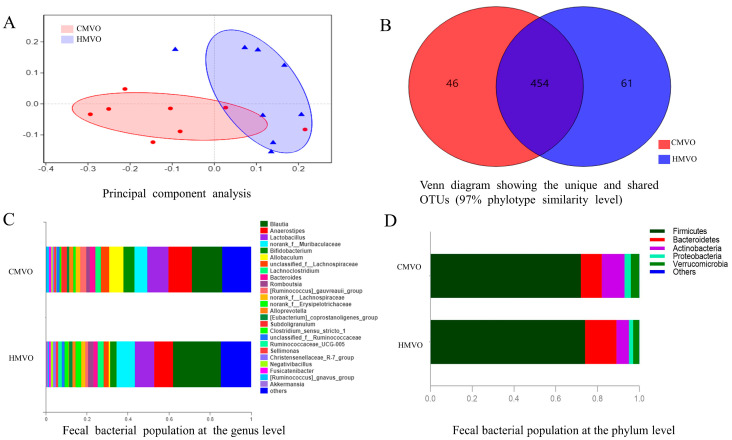
(**A**) shows the significant differences in the different groups through a principal component analysis; (**B**) shows the unique and shared OTUs in the different groups; (**C**) shows the fecal bacterial population at the phylum level in the different groups; and (**D**) shows the fecal bacterial population at the genus level in the different groups. HMVO = homemade mixed vegetable oil; CMVO = commercial mixed vegetable oil; OTUs: operational taxonomic units.

Alpha (α) diversity refers to the diversity within a particular region or ecosystem, including the Chao richness estimator index (Chao), the Sobs index (Sobs), the Shannon–Wiener diversity index (Shannon) and the Simpson diversity index (Simpson). Chao and Sobs both belong to operational taxonomic units (OUTs) [28]. In this study, the values of Chao and Sobs in the HMVO group were 326.75 and 384.58, respectively, which were significantly higher than those in the CMVO group (268.75 for Chao and 311.31 for Sobs, *p* < 0.05), indicating that the microbial richness was obviously affected by the HMVO when compared with the CMVO. Beta diversity analysis is used for species diversity through inter-group comparative analysis and further explores the similarity or difference between different microbial communities. It was evident that the intestinal flora did not overlap in the different groups according to the principal component analysis (PCA) (Figure 1A), indicating that the intestinal flora of the HMVO and CMVO groups was different, with an Anosim value of 0.2483.

#### 2.5.2. Species Composition Analysis

In order to know the similarity and overlap of the species compositions in the different samples, a Venn diagram can be used to count the number of common and unique species in multiple samples. It was evident that the number of OTUs in the HMVO and CMVO groups was 515 and 500, respectively (Figure 1B). A total of 454 OTUs in the rat fecal microflora were found in both the HMVO and CMVO groups, indicating that the microbial composition of these two groups was similar. Moreover, 46 and 61 unique OTUs were observed in the CMVO and HMVO groups, respectively. Figure 1C,D show the sample classification at the phylum and genus levels in the different groups. At the phylum classification level, firmicutes, bifidobacteria and actinobacteria were the most important microorganism species. The firmicutes and bifidobacteria were the dominant microorganisms in the HMVO and CMVO groups, whose percentages were more than 85% of the total microbial flora. Through the 16S r DNA gene method, genera were mainly detected in all the groups, and the blautia, anaerostipes, lactobacillus, muribaculaceae and bifidobacteria were the main bacteria with the highest abundances. Furthermore, the percentages of blautia and muribaculaceae in the CMVO group were lower than those in the HMVO group, while the mice fed with the HMVO had higher abundances of anaerostipes, lactobacillus and bifidobacteria. 

#### 2.5.3. Species Difference Analysis

**Figure 2:**
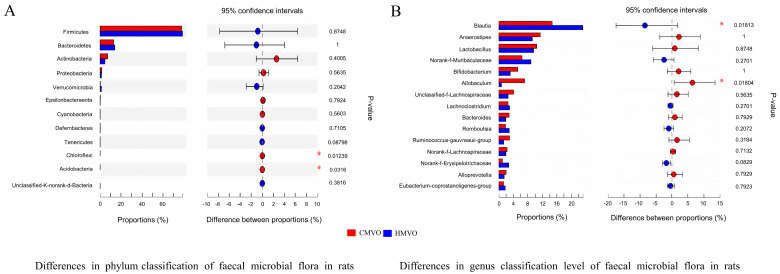
(**A**) shows the differences in the phylum classification of the fecal microbial flora in the different groups; (**B**) shows the differences in the genus classification level of the fecal microbial flora in the different groups. HMVO = homemade mixed vegetable oil; CMVO = commercial mixed vegetable oil; * means the significant difference between CMVO and HMVO.

There was no significant difference between the abundances of firmicutes, bacteroidetes, and actinobacteria in the HMVO and CMVO groups (Figure 2A,B). However, the abundances of chloroflexi and acidobacteria in the HMVO group were significantly higher compared with the CMVO group (*p* < 0.05), while the abundances of chloroflexi and acidobacteria in the HMVO group were lower compared with other microorganisms at the phylum level. At the genus level, the abundance of almost all microorganisms in the HMVO group was higher than that in the CMVO group, except allobacululum and subdoligranulum. The concentration of blautia, which can clear gas in the gut, was the most abundant. Another dominant bacteria was Ruminococcus, whose main fermentation metabolites were acetic acid and formic acid, and they mainly obtained energy by absorbing monosaccharides and degrading mucin. Therefore, based on the analyses of the different classification levels of the fecal microflora of the rats, the intestinal microflora of the rats fed the HMVO was more beneficial to the healthy development of the intestinal flora compared with the rats fed with the CMVO.

## 3. Materials and Methods

### 3.1. Materials

The vegetable oils (walnut oil, palm oil, linseed oil, soybean oil, rapeseed oil, sunflower oil and maize oil) were purchased from the local market, and the internal standard (1,3 (d 5)-diheptadecanoyl-2-heptadecenoyl-glycerol (d 5-(17:0/17:1/17:0) TAGs)) was obtained from Avanti Polar Lipids (Birmingham, AL, USA). Methanol (MeOH), hydrochloric acid (HCL), ethyl alcohol, n-Hexane, acetonitrile, isopropanol (IPA), chloroform (CHCl_3_), formic acid and ammonium formate were bought from Fisher Scientific (Pittsburgh, PA, USA). The standard mixture of 37 FA methyl esters (FAMEs) was purchased from ANPEL Laboratory Technologies Inc. (Shanghai, China). 

### 3.2. Fatty acid Analysis

The vegetable oils were weighed to 30 mg in a glass tube, and then n-Hexane (1 mL), methanol (2 mL) and solution (methanol/HCL, 3/1, *v*/*v*) were added and then shaken for 2 min. The tube was transferred to a water bath (100 °C) for 1 h, and it was cooled to room temperature after heating. After that, distilled water (2 mL) was pipetted and vortexed for 1 min, and then the tube was separated by centrifugation (1500× *g*, 5 min), and the organic liquid was obtained for gas chromatography analysis (GC, Agilent 8890B, Agilent Technologies Co., Ltd., Santa Clara, CA, USA). The specific procedure for the GC column (DB-23 60 m × 0.25 mm × 0.25 μm; Sigma-Aldrich, St. Louis, MI, USA) was taken from previous studies and modified. Briefly, the initial temperature was set at 50 °C (1 min) and then increased to 180 °C at a rate of 20 °C/min; after that, the temperature still increased to 230 at a rate of 1.3 °C/min and was kept constant for 5 min [29,30].

### 3.3. Triacylglycerol Analysis

All vegetable oils (5 mg) were dissolved in solution (CHCl_3_/methanol, 1/2, *v*/*v*) to 5 mg/mL, and then it was diluted (50 times) with methanol, which contained ammonium formate (10 mM), formic acid (0.1%) and internal standard (1,3(d5)-diheptadecanoyl-2-heptadecenoyl-glycerol (d5-(17:0/17:1/17:0) TAGs). For the setting condition of UPLC, mobile phase A was mixed with ACN and water (5:5, *v*/*v*), and mobile phase B was blended with IPA and ACN (9:1, *v*/*v*). They all contained ammonium formate (10 mM) and formic acid (0.1%). The chromatographic column was BEH C18 (1.7 μm, 2.1 mm ID × 100 mm, Waters Corporation, Milford, MA, USA), and the column temperature was 60 °C. The parameters of MS (4500QTrap, AB SCIEX, Framingham, MA, USA) are reported in detail in our previous works [3].

### 3.4. Establishment of Evaluation Model

#### 3.4.1. Determination of the Evaluation Conditions

The principle of the similarity evaluation model concerns the optimal value distance method, which is a type of comprehensive evaluation based on distance detection. The FA and TAG composition data of the HMF were obtained from our previous works (Table S1) [3]. For the first evaluation condition group, the TAGs were selected, including 18 kinds of TAG molecular species whose average percentages were higher than 1% of the total TAGs. The second evaluation condition group was FA species, a total of 13 FA species (the proportion of these FAs was higher than 1% of the total FAs), and some PUFAs, such as DHA. Moreover, it was reported that if C14:0, C16:0 and C18:0 were connected at the Sn-2 position, these FAs would be absorbed easily by infants, and feces hardening would be reduced. Therefore, the percentages of Sn-2 C14:0, Sn-2 C16:0 and Sn-2 C18:0 were regarded as the third evaluation condition group. Finally, some special structured TAGs, whose FA species connected at the Sn-1/3 position of the TAG, were easily absorbed and digested by infants. Therefore, the fourth evaluation condition group was the specially structured TAGs, including TAGs connected with three UFAs (UUU), TAGs connected with two UFAs at the Sn-1/3 position and one SFA at the Sn-2 position (USU), and other TAG-connected SFAs with carbon numbers less than 14 at the Sn-1/3 position (Table S1).

#### 3.4.2. Establishment of Evaluation Model

The similarity degree of the HMFS was assessed using the “deduction” principle. Due to the fact that TAGs account for a high proportion (98%) of the HMF, their highest simulation score was the highest, which was set at 50 points. The FAs were the basic components of the TAGs, so their highest simulation score was set at 25 points. The “Sn-2 FA” and the “special TAG structures” were the functional components for the development of the infants, so their highest simulation scores were set at 12.5 points and 12.5 points, respectively. 

Evaluation condition:

G = G1 + G2 + G3 + G4(1)

G1 = 50 − ∑Ei(TAG)(2)

G2 = 25 − ∑Ei(FA)(3)

G3 = 12.5 − ∑Ei(Sn-2 FA)(4)

G4 = 12.5 − ∑Ei(Special structure TAG)(5)

(6) $$\mathrm{Ei}=50\;\mathrm{Ei}=50\;(\mathrm{Ci}\frac{Di}{\sum Di})$$

(7) $$\mathrm{Ci}=\frac{\left|Bi-Ai\right|}{Ai}$$

G is the total score of the HMFS, with a highest score of 100; G1, G2, G3 and G4 are the scores of the TAGs, FAs, Sn-2 FAs, and special TAG structures, respectively, with highest scores of 50, 25, 12.5 and 12.5, respectively; Ei is the deducted score corresponding to each evaluation index; Ci is the floating coefficient of each evaluation index; Di is the average value of the corresponding evaluation index in the HMF; and Bi is the value of each evaluation index of the HMFS, mixed with different proportions of vegetable oils. Ai is the value of the range of each evaluation index of the HMF; when Bi is within the range of breast milk, Ai is 0; when Bi is higher than the maximum value of the evaluation index, Ai takes the maximum value of the evaluation index; and when Bi is lower than the minimum value of the evaluation index, Ai takes the minimum value of the evaluation index.

#### 3.4.3. Establishment of the Computer Software

The computer program was used to calculate the optimal formula for mixed vegetable oils, which was based on the C++ programming language. Three to five kinds of vegetable oils were selected, which is in accordance with commercial mixed vegetable oils. The minimum and maximum of each vegetable oil in each mixed formula were set at 0.001% and 100%, so the software was calculated from 0.001%, and the sum of the different vegetable oils was 100%. After obtaining a mixed vegetable oil, it was scored by the similarity evaluation model to determine the similarity between itself and the HMF.

### 3.5. In Vivo Evaluation of Nutritional Function

#### 3.5.1. Animals, Experiment Design and Treatments

Sixteen SD mice (male, 21–28-day-old at the start of the experiment, from Beijing Huafukang Bioscience CO., LTD., Beijing, China) were housed under a 12 h/12 h light/dark cycle at 22 ± 2 °C with 55 ± 5% humidity. These mice were randomly divided into two groups, namely the HMVO (*n* = 8) and CMVO (*n* = 8) groups. To adapt to the environment, these mice were fed with an AIN-93 diet one week before the experiment, and the feeding time was 28 days, during which the mice were free to eat and drink. Their body weight and food intake were recorded every 3 days, and the food utilization rate was recorded in grams of body weight increased per 100 g feed (g weight/100 g feed). On the 28th day, all rats were sacrificed by cervical dislocation. Intestinal contents, feces and colon tissue were immediately collected in sterile freezer tubes and frozen in liquid nitrogen for further analysis. All animal-related experiments were approved by the Animal Care and Use Committee of the Institute of Food Science and Technology, Chinese Academy of Agricultural Sciences (IFST-2022-124).

#### 3.5.2. Blood Routine and Serum Biochemical Indices 

On the last day of feeding, the mice fasted for 12 h, their blood was collected and the blood routine indices were determined with a blood cell counter: RBCs, HGB, HCT, MCH, MCV, MCHC, PLTs, WBCs, LYMs, NEUTs, MONOs, EOs, BAEOs and RETs. An automatic blood biochemical analyzer was used to detect the serum biochemical indices, including CHO, TG, LDL-c and HDL-c. 

#### 3.5.3. FA Profile of Intestinal Contents and Feces

The intestinal contents or feces (50 mg) and solution (12 mL, CHCl_3_/methanol, 2/1, *v*/*v*) were added into a tube, and they were shaken dramatically (2 min); after that, 4 mL of CHCl_3_ and 4 mL of distilled water were subsequently added and vortexed for 1 min. The mixing solution was centrifuged (3500× *g*, 10 min), and then organic liquid was obtained and dried by nitrogen concentration [31]. Finally, the methyl esterification of the FAs in the mice’s intestinal contents or feces was carried out according to the procedure in Section 3.2.

#### 3.5.4. Determination of Calcium in Feces

The feces were dried by freeze drying; they weighed about 10 mg and were transferred to a microwave digestion tank. Then, 5 mL of nitric acid was added, and it was left to stand for 1 h. Then, the tank was put under heated conditions (150–170 °C) for 4 h. After digestion, the sample was ultra-sounded for 5 min at 100 °C, and it was then filled to a constant volume (25 mL) for inductively coupled plasma mass spectrometry (ICP-MS). The specific parameters of the ICP-MS were as follows: The radio-frequency power was 1500 w and the flow of plasma, carrier gas, auxiliary gas and helium was 15 L/min, 0.8 L/min, 0.4 L/min and 4 mL/min, respectively. The temperature of the atomizing chamber was 2 °C, and the lifting rate was 0.3 r/s. 

#### 3.5.5. Metagenomic Detection of Intestinal Flora

A DNA extraction kit was used to extract DNA from fecal samples, and specific primers with “5 ‘barcode-primer-barcode 3’” were synthesized according to the specified sequencing region (V3–V4). The polymerase chain reaction products were quantified with a multifunctional enzyme marker, and then the DNA library was constructed and sequenced. The original data were filtered to obtain the optimized sequence. Afterward, the sequenced results were combined, and then they were analyzed via quantitative insights into the microbial ecology (QIIME) package, version 1.9.1, from two perspectives: an OUT cluster analysis and a taxonomic analysis. Based on the results of the OTU analysis, the diversity index and sequencing depth were detected, and a statistical analysis of the community structure was conducted at each taxonomic level. A consistency analysis was performed by combining the OTU and taxonomy analyses, and we further obtained the basic analysis results for the OTU of each group and its corresponding taxonomic lineage. 

### 3.6. Statistical Analysis

Each vegetable oil was bought repeatedly, and they were detected three times and expressed as mean values ± standard deviation. A one-way ANOVA was performed to analyze the data, and the significant differences among groups were determined by the Duncan multiple-range tests (SPSS 24.0 software, Chicago, IL, USA). GraphPad Prism 8 (San Diego, CA, USA) was used for figure processing.

## 4. Conclusions

In conclusion, 33 FA species and 63 TAG molecular species were detected in seven different vegetable oils, and C16:0, C18:1n9c, C18:2n6c, C18:3n6, 18:1/16:0/18:1, 18:2/16:0/18:2, 18:1/18:1/18:1, 18:1/18:2/18:1 and 18:2/18:0/18:2 were the main species among the FAs and TAGs in the vegetable oils. Through the software and similarity evaluation model, the optimal formula for mixed vegetable oils was composed of maize oil (43.4%), rapeseed oil (29.9%), palm oil (21.3%), linseed oil (2.8%) and soybean oil (2.6%), with the highest similarity score of 83.146. The in vivo experiment showed that the weight, blood routine indices and calcium and magnesium concentrations of the mice in the HMVO and CMVO groups were similar. Moreover, the percentages of C8:0 and C24:1 in the intestinal content and feces were lower in the HMVO group compared with the CMVO group. For the microbial composition, higher abundances of anaerostipes, lactobacillus and bifidobacterium were observed in the HMVO group. These results verified the practicability of the optimal mixed vegetable oils through the integration of the similarity evaluation model and computer software. Furthermore, we should focus on the optimization of the evaluation model to accelerate the development of HMFS in the future and find more evaluation methodologies to detect similarities between HMF and HMFS. 

## Figures and Tables

**Table 1:** The content of different FAs in vegetable oils (% is shown as the average ± standard deviation).

Fatty Acid	Walnut Oil	Plam Oil	Linseed Oil	Soybean Oil	Rapeseed Oil	Sunflower Oil	Maize Oil
C4:0	0.074 ± 0.016	0.665 ± 0.021	0.072 ± 0.024	0.096 ± 0.021	0.035 ± 0.008	0.037 ± 0.003	0.069 ± 0.016
C6:0	0.202 ± 0.026	0.161 ± 0.016	0.138 ± 0.032	0.176 ± 0.023	0.195 ± 0.027	0.153 ± 0.020	0.152 ± 0.020
C8:0	2.130 ± 0.087	1.857 ± 0.423	2.558 ± 0.360	2.174 ± 0.093	2.381 ± 0.120	2.512 ± 0.121	2.509 ± 0.113
C10:0	5.805 ± 1.886	4.536 ± 0.988	4.928 ± 2.116	5.049 ± 1.639	4.666 ± 1.533	4.897 ± 1.601	5.447 ± 1.774
C11:0	0.097 ± 0.006	0.075 ± 0.015	0.023 ± 0.004	0.069 ± 0.004	0.061 ± 0.004	-	0.106 ± 0.007
C12:0	0.064 ± 0.002	0.193 ± 0.047	-	0.078 ± 0.003	0.015 ± 0.001	-	-
C13:0	0.041 ± 0.001	0.055 ± 0.014	0.096 ± 0.011	0.063 ± 0.002	0.057 ± 0.002	0.302 ± 0.009	0.031 ± 0.001
C14:0	0.091 ± 0.002	1.088 ± 0.271	0.048 ± 0.006	0.076 ± 0.002	0.066 ± 0.002	0.115 ± 0.003	0.087 ± 0.002
C14:1	0.041 ± 0.001	-	-	-	-	-	-
C15:0	0.022 ± 0.000	0.046 ± 0.014	-	0.034 ± 0.000	0.023 ± 0.000	0.055 ± 0.001	-
C16:0	9.508 ± 0.034	37.093 ± 10.59	6.117 ± 0.560	10.634 ± 0.013	4.670 ± 0.031	6.576 ± 0.028	12.923 ± 0.011
C16:1	0.112 ± 0.000	0.219 ± 0.063	0.114 ± 0.011	0.117 ± 0.000	0.175 ± 0.002	0.075 ± 0.000	0.154 ± 0.001
C17:0	0.092 ± 0.005	0.136 ± 0.027	0.112 ± 0.016	0.163 ± 0.009	0.075 ± 0.005	0.030 ± 0.002	0.132 ± 0.007
C17:1	0.118 ± 0.002	0.033 ± 0.010	0.042 ± 0.004	0.070 ± 0.001	0.055 ± 0.000	0.051 ± 0.001	0.024 ± 0.000
C18:0	3.655 ± 0.067	4.397 ± 1.356	4.100 ± 0.308	4.315 ± 0.071	2.034 ± 0.017	3.866 ± 0.043	1.915 ± 0.028
C18:1n9t	0.028 ± 0.002	0.050 ± 0.020	0.023 ± 0.001	0.025 ± 0.002	0.025 ± 0.002	0.058 ± 0.004	0.103 ± 0.007
C18:1n9c	21.285 ± 0.867	37.63 ± 15.567	19.692 ± 9.669	19.483 ± 0.740	53.225 ± 1.581	18.917 ± 0.609	26.843 ± 0.961
C18:2n6t	-	-	0.870 ± 0.113	1.377 ± 0.049	3.338 ± 0.144	-	-
C18:2n6c	49.671 ± 1.147	10.704 ± 3.366	15.736 ± 1.121	48.459 ± 0.994	20.063 ± 0.249	61.268 ± 0.913	48.049 ± 0.883
C20:0	0.155 ± 0.039	0.230 ± 0.154	0.517 ± 0.084	0.211 ± 0.052	0.465 ± 0.109	0.163 ± 0.038	0.234 ± 0.057
C18:3n6	6.041 ± 0.121	0.263 ± 0.066	44.235 ± 5.098	6.624 ± 0.147	6.307 ± 0.188	0.142 ± 0.003	0.600 ± 0.015
C20:1n9	0.205 ± 0.000	0.140 ± 0.000	0.177 ± 0.012	0.171 ± 0.004	1.147 ± 0.018	0.119 ± 0.002	0.202 ± 0.004
C18:3n3	0.061 ± 0.001	-	0.069 ± 0.000	0.026 ± 0.000	0.061 ± 0.002	-	0.084 ± 0.001
C21:0	0.017 ± 0.000	0.052 ± 0.015	-	0.047 ± 0.000	0.057 ± 0.000	0.023 ± 0.001	0.026 ± 0.000
C20:2	0.166 ± 0.000	0.035 ± 0.04	0.120 ± 0.056	0.223 ± 0.122	0.162 ± 0.087	0.359 ± 0.193	0.056 ± 0.031
C22:0	-	-	-	-	0.194 ± 0.000	-	-
C20:3n6	0.021 ± 0.001	-	-	-	-	-	0.057 ± 0.000
C22:1	-	0.077 ± 0.012	-	0.027 ± 0.002	-	-	-
C20:3n6	-	-	0.082 ± 0.001	-	0.114 ± 0.012	-	-
C20:4n6	0.063 ± 0.000	0.054 ± 0.015	0.020 ± 0.002	0.058 ± 0.001	0.027 ± 0.001	0.051 ± 0.001	0.017 ± 0.000
C24:0	0.121 ± 0.005	0.098 ± 0.020	0.112 ± 0.015	0.156 ± 0.006	0.166 ± 0.008	0.231 ± 0.010	0.155 ± 0.006
C24:1	0.038 ± 0.000	-	-	-	0.141 ± 0.002	-	-
C22:6	0.078 ± 0.002	0.113 ± 0.027	-	-	-	-	0.026 ± 0.001

**Table 2:** The contents of different TAGs in vegetable oil (% is shown as the average ± standard deviation).

TAG	Abbreviation	Walnut Oil	Plam Oil	Linseed Oil	Soybean Oil	Rapeseed Oil	Sunflower Oil	Maize Oil
12:0/10:0/8:0	LaCCy	0.002 ± 0.000 ^a^	0.002 ± 0.001	-	-	-	-	-
12:0/6:0/12:0	LaCaLa	-	0.002 ± 0.000	-	-	-	-	-
12:0/8:0/12:0	LaCyLa	-	0.019 ± 0.012	-	-	-	-	-
12:0/6:0/14:0	LaCaM	0.001 ± 0.000	0.002 ± 0.000	-	-	-	-	-
12:0/8:0/14:0	LaCyM	0.008 ± 0.003	0.013 ± 0.003	0.006 ± 0.001	-	0.004 ± 0.001	-	-
12:0/10:0/12:0	LaCLa	0.005 ± 0.001	0.016 ± 0.003	-	-	-	-	-
12:0/12:0/12:0	LaLaLa	0.025 ± 0.005	0.203 ± 0.050	0.019 ± 0.004	0.003 ± 0.001	0.009 ± 0.003	0.002 ± 0.000	0.002 ± 0.000
12:0/10:0/14:0	LaCM	0.004 ± 0.001	0.011 ± 0.002	-	-	0.003 ± 0000	-	-
14:0/8:0/14:0	MCyM	0.005 ± 0.000	0.014 ± 0.003	0.008 ± 0.002	-	0.003 ± 0.001	-	-
12:0/14:0/12:0	LaMLa	0.018 ± 0.004	0.115 ± 0.013	0.007 ± 0.001	-	0.007 ± 0.001	0.001 ± 0.000	-
12:0/14:0/14:0	LaMM	0.01 ± 0.001	0.033 ± 0.007	0.010 ± 0.003	-	0.006 ± 0.001	-	-
12:0/16:0/12:0	LaPLa	0.008 ± 0.002	0.031 ± 0.007	0.014 ± 0.003	-	0.009 ± 0.002	-	-
14:0/16:0/12:0	MPLa	0.004 ± 0.001	0.019 ± 0.003	-	-	0.005 ± 0000	-	-
12:0/18:0/12:0	LaSLa	0.004 ± 0.000	0.009 ± 0.001	-	-	0.002 ± 0000	-	-
14:0/14:0/14:0	MMM	0.005 ± 0.001	0.02 ± 0.004	-	-	0.006 ± 0000	-	-
14:0/16:0/14:0	MPM	-	0.024 ± 0.002	-	-	-	-	-
16:0/12:0/16:0	PLaP	-	0.037 ± 0.004	-	-	-	-	-
16:0/14:0/16:0	PMP	-	0.096 ± 0.01	-	-	-	-	-
16:0/12:0/18:0	PLaS	-	0.007 ± 0.000	-	-	-	-	-
14:0/18:2/16:0	MLP	0.041 ± 0.005	0.346 ± 0.014	0.014 ± 0.001	0.059 ± 0.004	0.026 ± 0.002	0.039 ± 0.002	0.025 ± 0.002
16:0/18:0/16:0	PSP	0.040 ± 0.002	0.059 ± 0.001	0.113 ± 0.004	0.044 ± 0.002	0.019 ± 0.004	0.029 ± 0.001	0.033 ± 0.001
16:0/18:1/16:0	POP	1.738 ± 0.083	38.714 ± 1.722	1.737 ± 0.076	1.995 ± 0.062	0.930 ± 0.046	0.801 ± 0.024	3.073 ± 0.114
18:1/14:0/18:1	OMO	0.070 ± 0.003	0.664 ± 0.009	0.117 ± 0.006	0.078 ± 0.004	0.197 ± 0.013	0.056 ± 0.002	0.069 ± 0.004
16:0/18:2/16:0	PLP	4.051 ± 0.204	13.350 ± 1.626	1.723 ± 0.100	5.39 ± 0.214	0.87 ± 0.038	2.733 ± 0.151	6.74 ± 0.254
18:2/16:1/16:0	LPoP	0.324 ± 0.019	0.147 ± 0.006	1.884 ± 0.092	0.401 ± 0.013	0.193 ± 0.014	0.049 ± 0.002	0.116 ± 0.006
18:2/18:1/14:0	LOM	0.044 ± 0.002	0.085 ± 0.003	0.035 ± 0.002	0.046 ± 0.002	0.043 ± 0.009	0.058 ± 0.003	0.024 ± 0.001
16:0/18:3/16:0	PLnP	0.121 ± 0.005	0.063 ± 0.002	0.831 ± 0.042	0.155 ± 0.005	0.06 ± 0.008	0.003 ± 0.000	0.026 ± 0.001
18:0/16:0/18:0	SPS	0.022 ± 0.001	-	0.089 ± 0.003	0.034 ± 0.002	0.027 ± 0.011	0.019 ± 0.001	0.01 ± 0.001
18:1/12:0/18:1	OLaO	-	0.042 ± 0.002	-	-	0.040 ± 0.000	-	-
16:0/18:1/18:0	POS	0.505 ± 0.037	3.752 ± 0.268	0.917 ± 0.012	0.686 ± 0.031	0.243 ± 0.004	0.275 ± 0.018	0.312 ± 0.027
18:1/16:0/18:1	OPO	4.351 ± 0.159	21.965 ± 0.264	6.524 ± 0.273	4.226 ± 0.32	8.552 ± 0.167	2.841 ± 0.025	5.857 ± 0.316
18:2/18:0/16:0	LSP	0.764 ± 0.024	0.826 ± 0.038	0.474 ± 0.042	1.113 ± 0.059	0.145 ± 0.003	0.701 ± 0.039	0.451 ± 0.029
18:1/16:1/18:1	OPoO	5.739 ± 0.272	4.162 ± 0.348	3.383 ± 0.136	6.492 ± 0.488	3.52 ± 0.068	4.868 ± 0.048	8.432 ± 0.094
18:1/16:0/18:2	OPL	5.073 ± 0.081	3.652 ± 0.190	3.148 ± 0.605	5.802 ± 0.166	2.709 ± 0.076	4.563 ± 0.396	7.778 ± 0.358
18:2/16:0/18:2	LPL	11.273 ± 2.406	1.088 ± 0.031	3.353 ± 0.057	12.172 ± 1.369	1.309 ± 0.027	10.959 ± 0.222	12.538 ± 1.204
18:2/16:1/18:2	LPoL	1.325 ± 0.071	0.025 ± 0.003	4.647 ± 0.307	1.341 ± 0.057	0.416 ± 0.012	0.204 ± 0.012	0.263 ± 0.007
18:3/16:0/18:2	LnPL	1.372 ± 0.030	0.03 ± 0.002	5.576 ± 0.188	1.526 ± 0.015	0.398 ± 0.019	0.038 ± 0.003	0.173 ± 0.012
18:0/18:1/18:0	SOS	0.290 ± 0.010	0.402 ± 0.046	0.913 ± 0.054	0.416 ± 0.040	0.165 ± 0.012	0.163 ± 0.015	0.079 ± 0.008
20:0/18:1/16:0	AOP	0.103 ± 0.012	0.377 ± 0.059	0.124 ± 0.014	0.101 ± 0.010	0.101 ± 0.008	0.049 ± 0.004	0.112 ± 0.014
18:1/18:0/18:1	OSO	1.314 ± 0.147	1.840 ± 0.154	3.164 ± 0.273	1.460 ± 0.185	2.327 ± 0.135	1.021 ± 0.093	0.634 ± 0.023
18:0/18:2/18:0	SLS	1.250 ± 0.105	1.392 ± 0.131	2.584 ± 0.207	1.440 ± 0.176	1.709 ± 0.049	1.009 ± 0.122	0.530 ± 0.06
16:0/20:0/18:2	PAL	0.110 ± 0.005	0.081 ± 0.009	0.122 ± 0.015	0.188 ± 0.016	0.152 ± 0.012	0.071 ± 0.007	0.160 ± 0.013
18:1/18:1/18:1	OOO	4.164 ± 0.352	2.690 ± 0.451	8.485 ± 0.678	3.892 ± 0.508	26.833 ± 1.226	4.928 ± 0.205	4.257 ± 0.456
18:1/18:2/18:0	OLS	2.048 ± 0.044	0.524 ± 0.047	1.141 ± 0.074	2.466 ± 0.211	1.205 ± 0.108	1.918 ± 0.132	0.936 ± 0.104
18:1/18:2/18:1	OLO	10.805 ± 0.097	1.058 ± 0.162	13.194 ± 1.181	9.323 ± 0.898	22.025 ± 1.301	11.285 ± 0.717	12.551 ± 1.501
18:2/18:0/18:2	LSL	9.632 ± 1.027	0.672 ± 0.089	6.817 ± 0.465	9.284 ± 0.312	11.601 ± 1.000	11.598 ± 0.386	8.454 ± 0.589
18:2/18:1/18:2	LOL	10.012 ± 0.222	0.181 ± 0.013	5.649 ± 0.387	9.628 ± 0.450	4.149 ± 0.254	16.181 ± 1.053	12.001 ± 0.601
18:2/18:2/18:2	LLL	16.676 ± 0.18	0.070 ± 0.009	10.835 ± 1.571	14.753 ± 0.475	2.741 ± 0.227	20.694 ± 2.148	11.618 ± 0.099
18:2/18:3/18:2	LLnL	3.744 ± 0.303	0.010 ± 0.001	9.664 ± 1.086	3.284 ± 0.360	0.603 ± 0.020	0.078 ± 0.008	0.356 ± 0.025
22:0/18:1/16:0	DOP	0.107 ± 0.010	0.105 ± 0.019	0.202 ± 0.033	0.116 ± 0.020	0.096 ± 0.008	0.090 ± 0.014	0.070 ± 0.012
18:1/20:0/18:1	OAO	0.236 ± 0.031	0.287 ± 0.044	0.632 ± 0.068	0.251 ± 0.024	1.253 ± 0.131	0.207 ± 0.018	0.256 ± 0.037
16:0/22:0/18:2	PDL	0.155 ± 0.023	0.036 ± 0.006	0.122 ± 0.013	0.217 ± 0.024	0.133 ± 0.009	0.203 ± 0.025	0.064 ± 0.007
18:1/20:1/18:1	OGO	0.194 ± 0.022	0.085 ± 0.013	0.351 ± 0.040	0.215 ± 0.032	1.741 ± 0.185	0.141 ± 0.018	0.260 ± 0.033
18:2/18:1/20:0	LOA	0.168 ± 0.018	0.061 ± 0.008	0.117 ± 0.011	0.227 ± 0.034	0.330 ± 0.035	0.154 ± 0.018	0.206 ± 0.018
18:2/20:1/18:1	LGO	0.106 ± 0.014	0.024 ± 0.004	0.303 ± 0.031	0.133 ± 0.022	0.821 ± 0.112	0.081 ± 0.008	0.146 ± 0.013
18:2/20:0/18:2	LAL	0.285 ± 0.036	0.055 ± 0.006	0.199 ± 0.022	0.381 ± 0.061	0.645 ± 0.085	0.385 ± 0.037	0.400 ± 0.048
18:2/20:1/18:2	LGL	0.193 ± 0.023	0.010 ± 0.002	0.161 ± 0.020	0.200 ± 0.030	0.209 ± 0.020	0.195 ± 0.014	0.241 ± 0.018
24:0/16:0/18:1	TPO	0.037 ± 0.005	0.080 ± 0.017	0.148 ± 0.018	0.038 ± 0.008	0.032 ± 0.005	0.041 ± 0.007	0.051 ± 0.007
18:1/22:0/18:1	ODO	0.209 ± 0.033	0.161 ± 0.036	0.372 ± 0.052	0.220 ± 0.045	0.537 ± 0.077	0.416 ± 0.080	0.129 ± 0.017
18:2/24:0/16:0	LTP	0.069 ± 0.009	0.026 ± 0.005	0.080 ± 0.012	0.097 ± 0.020	0.035 ± 0.005	0.080 ± 0.015	0.055 ± 0.010
18:1/22:1/18:1	ODoO	0.142 ± 0.017	0.038 ± 0.008	0.305 ± 0.037	0.207 ± 0.035	0.449 ± 0.082	0.338 ± 0.064	0.065 ± 0.012
22:0/18:1/18:2	DOL	0.148 ± 0.025	-	-	0.246 ± 0.034	0.182 ± 0.030	0.415 ± 0.073	0.065 ± 0.008
18:2/22:0/18:2	LDL	0.224 ± 0.025	-	-	0.329 ± 0.056	0.163 ± 0.021	0.793 ± 0.092	0.105 ± 0.013

^a^ The values represent the means ± SD. The abbreviations used are as follows: Ca, caproic acid, 6:0; Cy, caprylic acid, 8:0; C, capric acid, 10:0; La, lauric acid, 12:0; M, myristic acid, 14; P, palmitic acid, 16:0; Po, pamitoleic acid, 16:1; S, stearic acid, 18:0; O, oleic acid, 18:1; L, linoleic acid, 18:2; Ln, linolenic acid, 18:3; A, arachidic acid, 20:0; E, eicosenoic acid, 20:1; D, docosanoic acid, 22:0; Do, docosenoic acid, 22:1; T, tetracosanoic acid, 24:0.

**Table 3:** The calculation process for the optimal formula obtained by the evaluation model.

Conditions		Ci ^a^	Ei	∑Di	∑Ei	G
G 1	18:1/16:0/18:1	0.000	0.001	83.318	16.854	83.146
	18:1/16:0/18:2	0.296	2.007			
	18:1/18:1/18:1	0.000	0.000			
	18:1/18:2/18:1	0.000	0.000			
	18:2/16:0/18:2	0.000	0.000			
	16:0/18:1/16:0	0.104	0.318			
	18:2/18:1/18:2	0.000	0.000			
	18:1/14:0/18:1	0.874	2.081			
	16:0/18:2/16:0	0.135	0.279			
	18:0/16:0/18:1	1.000	1.955			
	18:1/12:0/18:1	0.977	1.444			
	18:1/18:0/18:1	0.000	0.000			
	18:1/16:0/12:0	1.000	1.404			
	18:1/16:0/14:0	1.000	1.178			
	18:2/18:2/18:2	0.000	0.000			
	18:2/14:0/18:1	1.000	0.793			
	18:1/16:0/16:1	1.000	0.682			
	18:1/12:0/18:2	1.000	0.658			
G 2	C10:0	1.067	0.412	94.468		
	C12:0	0.977	1.255			
	C14:0	0.834	0.822			
	C16:0	0.000	0.000			
	C18:0	0.245	0.341			
	C22:0	0.925	0.513			
	C16:1	0.854	0.41			
	C18:1	0.000	0.000			
	C18:2	0.000	0.000			
	C18:3	0.002	0.001			
	C20:4	0.000	0.000			
	C20:5	1.000	0.017			
	C22:6	0.753	0.067			
G 3	Sn-2	0.000	0.000			
	Sn-2	0.000	0.000	183.917		
	Sn-2	0.069	0.215			
G 4	UUU + USU	0.000	0.000	152.43		
	TAG with SFAs (carbon number < 14) at Sn-1/3 position	0.000	0.000			

^a^ G is the total score for the HMFS, with a highest score of 100; G1, G2, G3 and G4 are the scores for the TAGs, FAs, Sn-2 FAs and special TAG structures, respectively, with a highest score of 50, 25, 12.5 and 12.5, respectively; ∑Ei is the sum of Ei, which is the deducted score corresponding to each evaluation index; Ci is the floating coefficient of each evaluation index; ∑Di is the sum of Di, which is the average value of each corresponding evaluation index in the HMF. TAG, triglyceride; USU, TAG connected with three unsaturated fatty acids; USU, TAG connected with two unsaturated fatty acids at the Sn-1/3 position and one saturated fatty acid at the Sn-2 position; SFA, saturated fatty acid.

## Data Availability

The raw data supporting the conclusions of this article will be made available by the authors on request.

## Supplementary Materials

The following supporting information can be downloaded at https://www.mdpi.com/article/10.3390/molecules29092096/s1: Tables S1–S6: Conditions of mixed plant oil to mimic human milk; Top 1000 formulas output by the software; Food utilization and body weight of SD rats during the experiment; Results of routine blood and blood biochemistry test in each group; Fatty acids of contents in feces and small intestine of SD rats (%); The concentration of calcium and magnesium in each group (mg/g).
